# The complete chloroplast genome sequence of *Urtica fissa*

**DOI:** 10.1080/23802359.2022.2080017

**Published:** 2022-06-16

**Authors:** Kuiyin Li, Hailing Zhang, Miaoxiao Shi, Yubo Zhang, Chunlei Cong, Xiangcai Chang, Lili Duan, Yanqing Ding

**Affiliations:** a College of Agriculture, Guizhou University, Guiyang, P. R. China; b College of Agriculture, Anshun University, Anshun, P. R. China; c Institute of Upland Food Crops, Guizhou Academy of Agricultural Sciences, Guiyang, P. R. China

**Keywords:** *Urtica fissa*, chloroplast genome, phylogeny

## Abstract

*Urtica fissa* E. Pritz is not only an important medicinal plant for rheumatism and cough relief, but it is also an important forage plant. In this study, the complete chloroplast genome of *U. fissa* was assembled for the first time and reported to be 146,837 base pairs (bp) long with a typical tetragonal structure and including a large single-copy of 79,657 bp, a small single-copy of 17,712 bp, and two inverted repeats of 24,734 bp each. It harbors 115 unique genes, including 70 protein-coding genes, 38 transfer RNA genes, and 7 ribosomal RNA genes. Phylogenetic analysis showed that *U. fissa* is closely related to *Urtica lobatifolia*. This study contributes to the understanding of the origin and evolution of *U. fissa*, as well as its genetic relationships with other species.

The Urticaceae family consists of approximately 54 genera with more than 2000 species (Wu et al. 2013). *Urtica fissa* E. Pritz. 1900 (nettle), a species in the Urticaceae family, is an annual or perennial shrub armed with little stinging hairs. It is usually located in China and Vietnam at altitudes ranging from 100 to 2000 m (Editorial Committee of Flora of China, Chinese Academy of Sciences 1985). The leaves of *U. fissa* are commonly used to treat rheumatism, arthritis, and urticaria. Many chemical compounds with anti-inflammatory properties have recently been identified (Wang et al. 2012; Wang et al. 2018). The phylogenetic relationship among Urticaceae species has not been established to date. Illumina genome sequencing technology was used to further clarify the use of *U. fissa* and to establish its position in the Urticaceae species. The sequencing data was assembled and the complete chloroplast genome of *U. fissa* (GenBank accession number: MZ313540.1) was annotated. Total genomic DNA was extracted from fresh *U. fissa* leaves. The leaves were collected from plants grown in Guiyang, Guizhou Province, China (N 26°26′54.69′′, E 106°38′51.42′′). The specimens were placed at the Guizhou University herbarium under the voucher number ASXYnxy20210129 (http://agr.gzu.edu.cn/, Songshu Chen and sschen1@gzu.edu.cn). Chloroplast genome sequencing was performed on the NovaSeq 6000 sequencing system (Illumina, San Diego, CA, USA) at Wuhan Bena Biotechnology Co. Ltd. The complete chloroplast genome was assembled *de novo* using the GetOrganelle software (version: v1.7.3.5) (Jin et al. 2020). Gene annotation was performed using Plastid Genome Annotator (PGA, parameter: -i 1000 -p 40 -q 0.5, 2) (Qu et al. 2019). The sequences were aligned using MAFFT 7 (Katoh and Standley 2013) with conserved sequences selected using Gblocks (Talavera and Castresana 2007). Maximum-likelihood phylogenetic trees were generated using MEGA X (Kumar et al. 2018). Bootstrap values were calculated from 1000 replicates (Figure 1).

The complete genome of *U. fissa* (MZ313540.1), 146,837 bp in length with a GC content of 36.6%, possessed a typical tetragonal structure. This included a large single-copy of 79,657 bp with a GC content of 34.2%, a small single-copy region of 17,712 bp with a GC content of 30.2%, and two inverted repeats of 24,734 bp, both with GC contents of 42.8%. The genome consisted of 115 genes, including 70 protein-coding genes, 38 transfer RNA genes, and 7 ribosomal RNA genes. Phylogenetic analysis was performed on the complete chloroplast genomes of *U. fissa* and other related species in the Urticaceae family, with *Humulus lupulus* and *Cannabis sativa* in the Cannabaceae as outgroups. The phylogenetic tree showed that all Urtica species formed monophyletic branches. *Urtica fissa* E. Pritz was mostly related to *Urtica lobatifolia*, with a bootstrap support value of 100%. *Urtica fissa* was closely related to *U. lobatifolia* by phylogenetic analysis. This study adds to our understanding of the origin and evolution of *U. fissa*, as well as its genetic links with other species.

## Ethical approval and consent to participate

This article does not involve any studies with human or animal participants. The methods used in the study were performed according to the relevant guidelines and regulations. All experimental protocols were approved by Guizhou University. *Urtica fissa* is not on the list of National Important Wild Plants, and the sampling site is not within the National Nature Reserve.[Fig F0001]

## Authors’ contributions

KYL planned and designed the protocol. HLZ and MXS wrote the manuscript. LLD collected the samples, extracted the DNA, and sent the DNA for sequencing externally. YQD and CLC analyzed the data and assembled the chloroplast genome. YBZ and XCC revised the manuscript. All authors read and approved the final manuscript.

## Supplementary Material

Supplemental Material

Supplemental Material

## Data Availability

The genome sequence data of this study are available in GenBank (https://www.ncbi.nlm.nih.gov/) under accession no. MZ313540.1. The associated BioProject, SRA, and Bio-Sample numbers are PRJNA782209, SRR16991884, and SAMN23342227, respectively. Maximum-likelihood phylogenetic tree analysis based on chloroplast genome sequences, including the *U. fissa* sequenced in this study. The numbers on each node were assessed by bootstrapping from 1000 replicates.
