# Supplementary material for: The complete chloroplast genome sequence of Urtica fissa
Source: Mitochondrial DNA B Resour. 2022 Jun 16;7(6):1005–7. doi: 10.1080/23802359.2022.2080017 (PMC9225764; doi:10.1080/23802359.2022.2080017)
Supplement: Supplemental Material [file TMDN_A_2080017_SM7737.pdf]

CERTIFICATE OF ENGLISH EDITING

This document certifies that the paper listed below has been edited to ensure that the language is clear and free of errors. The edit was performed by professional editors at Editage, a division of Cactus Communications, in cooperation with Taylor & Francis Group. The intent of the author's message was not altered in any way during the editing process. The quality of the edit has been guaranteed, with the assumption that our suggested changes have been accepted and have not been further altered without the knowledge of our editors.

Title

The complete chloroplast genome sequence of Urtica fissa

Authors

Kuiyin Li, Hailing Zhang, Miaoxiao Shi, Yubo Zhang, Chunlei Cong, Xiangcai Chang, Lili Duan, Yanqing Ding

Order No.

DZOEY_1

EDITINGSERVICES
Supporting Taylor & Francis authors

Signature

Vikas Narang

Vikas Narang,
Chief Operating Officer,
Editage

Date of Issue
February 25, 2022
